# Supplementary material for: Potassium channels mediate the inhibitory effect of exosomes on anti-tumor immunity in head and neck cancer
Source: bioRxiv. 2025 Jul 28:2025.07.25.666838. Preprint. [Version 1] doi: 10.1101/2025.07.25.666838 (PMC12324221; doi:10.1101/2025.07.25.666838)
Supplement: Supplement 1 [file media-1.pdf]

Supplementary Materials:

Supplementary Methods:

Cal27 cell culture

Cal27 cells were obtained from American Tissue Culture Collection (ATCC, Manassas, VA, USA), grown as described previously in DMEM with 10% FBS, 1% penicillin–streptomycin, 1% minimum essential amino acids, 6 mM L-glutamine, and 1% sodium pyruvate in the presence of 5% CO2 at 37 °C in a humidified incubator (21). Mycoplasma testing was performed routinely using MycoStrip® mycoplasma detection kit (Invivogen, Catalog# rep-mys-50) and was negative for all cultures. PDL-1 knockout Cal 27 cell lines (PDL1-KO) were generated with CRISPR-Cas9 system at Transgenic Animal and Genome Editing (TAGE) core at CCHMC. Knockouts were validated by the core using RT-PCR. For the final two passages before exosome isolation, cells were cultured in media with 5% exosome-depleted FBS. Exosomes were isolated from the conditioned media of these cultured cells by ultracentrifugation (see Materials and Methods), and characterized as described previously (21).

On-bead flow cytometry.

Surface marker expression on exosomes was analyzed using on-bead flow cytometry as detailed previously (21). Exosomes (20 µg protein, measured with Qubit Assay, as described in Materials and Methods) were captured on anti-CD63 antibody-coated Dynabeads (Thermo Fisher) overnight at 4°C in isolation buffer (PBS + 0.1% BSA, 0.22 µm-filtered). Exosomes were stained with PE-conjugated antibodies against CD63 (RRID: AB_10896786), CD9 (RRID: AB_2075893), and CD81 (RRID: AB_10642024) (BioLegend). Controls included PE-conjugated IgG (RRID: AB_326435) and unstained beads. Data were acquired on a BD LSRFortessa cytometer and analyzed using FlowJo software (BD Biosciences).

Supplementary Figures and Tables

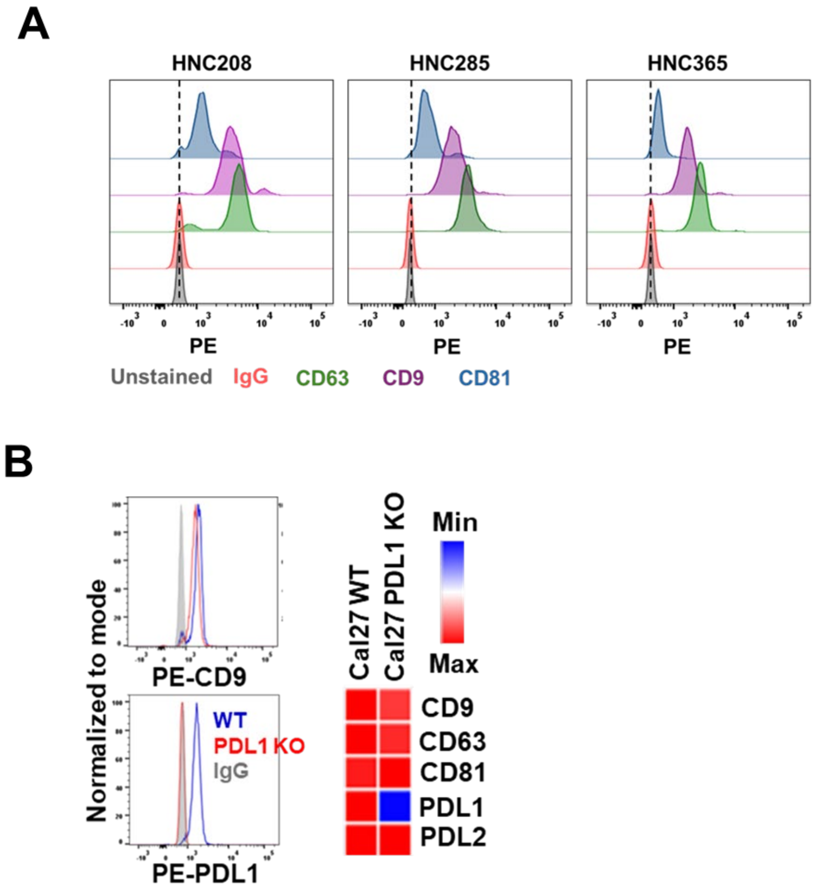

Fig. S1 Validation of exosome characterization methodology (A) Staggered histograms showing CD63, CD9, and CD81 abundance in exosomes isolated from HNSCC cell conditioned media that were captured on CD63-antibody-coated beads. Unstained and isotype control (IgG) stained beads were used as negative controls. (B) (Left) Overlay histograms showing CD9 (Top) and PDL1 (bottom) abundance in exosomes isolated from wild type (WT) and PDL1 knockout (PDL1-KO) Cal 27 cells that were captured on CD63-antibody-coated beads. Isotype control (IgG) stained beads were used as negative controls. (Right) Heatmap showing surface protein phenotyping of exosomes from WT and PDL1-KO Cal27 cells showing the abundance of tetraspanins (CD9, CD63 and CD81) and PD-1 ligands (PDL-1 and PDL-2) on intact exosome surface by multiplexed ELISA. Exosomes were captured by antibodies and detected by a signal from a cocktail of anti-tetraspanin antibodies (CD63, CD81, and CD9). Each sample was run in duplicate on the same plate.

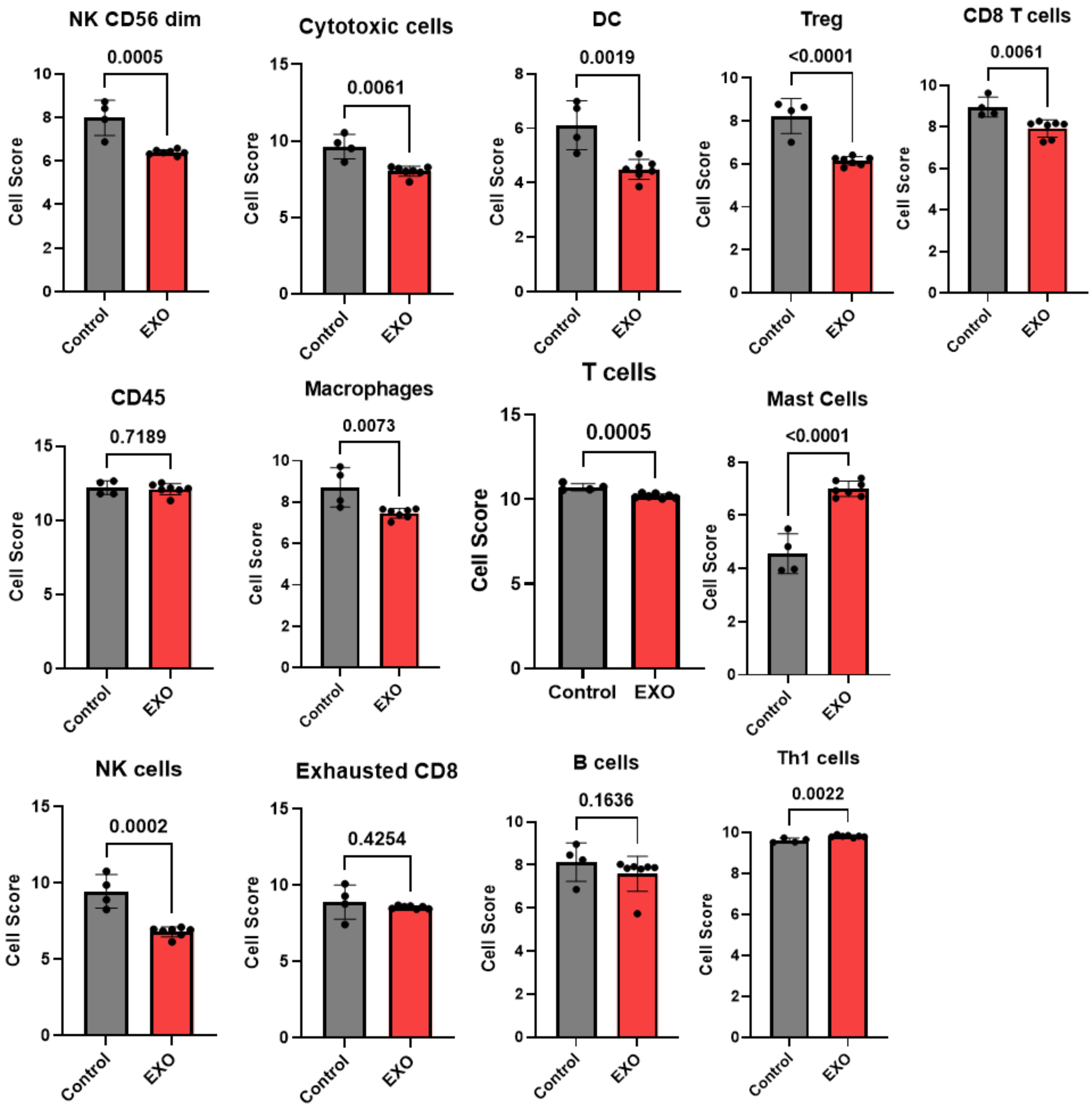

Fig. S2 Immune Cell Type Scores in PBMCs in the absence or presence of HNSCC exosomes Cell type scores from NanoString transcriptomic data showing immune cell abundance in PBMCs in the absence (Control) or presence of HNSCC exosomes (EXO). from untreated healthy donors (n=4; Control) and from healthy donors treated with exosomes (n=7; EXO) at a concentration of 1.04×10⁹ particles/ml. The EXO group included PBMCs from four healthy donors treated with exosomes derived from three different HNSCC patients (HNC208, HNC285, and HNC365); three of the donors were each treated with exosomes from two different patients. Significance between groups was determined by either Mann-Whitney rank sum test

(cytotoxic cells, B cells and CD8 T cells) or unpaired t-test. Bars represent means ± SD, symbols represent individual treatment conditions. The abundance of the different immune cell types (at the RNA level) in the various patient cohorts was calculated as log2 cell type scores. The cell scores for a specific cell type can only be compared between two groups (control vs EXO) but do not support claims that a cell type is more abundant than another cell type within the same group.

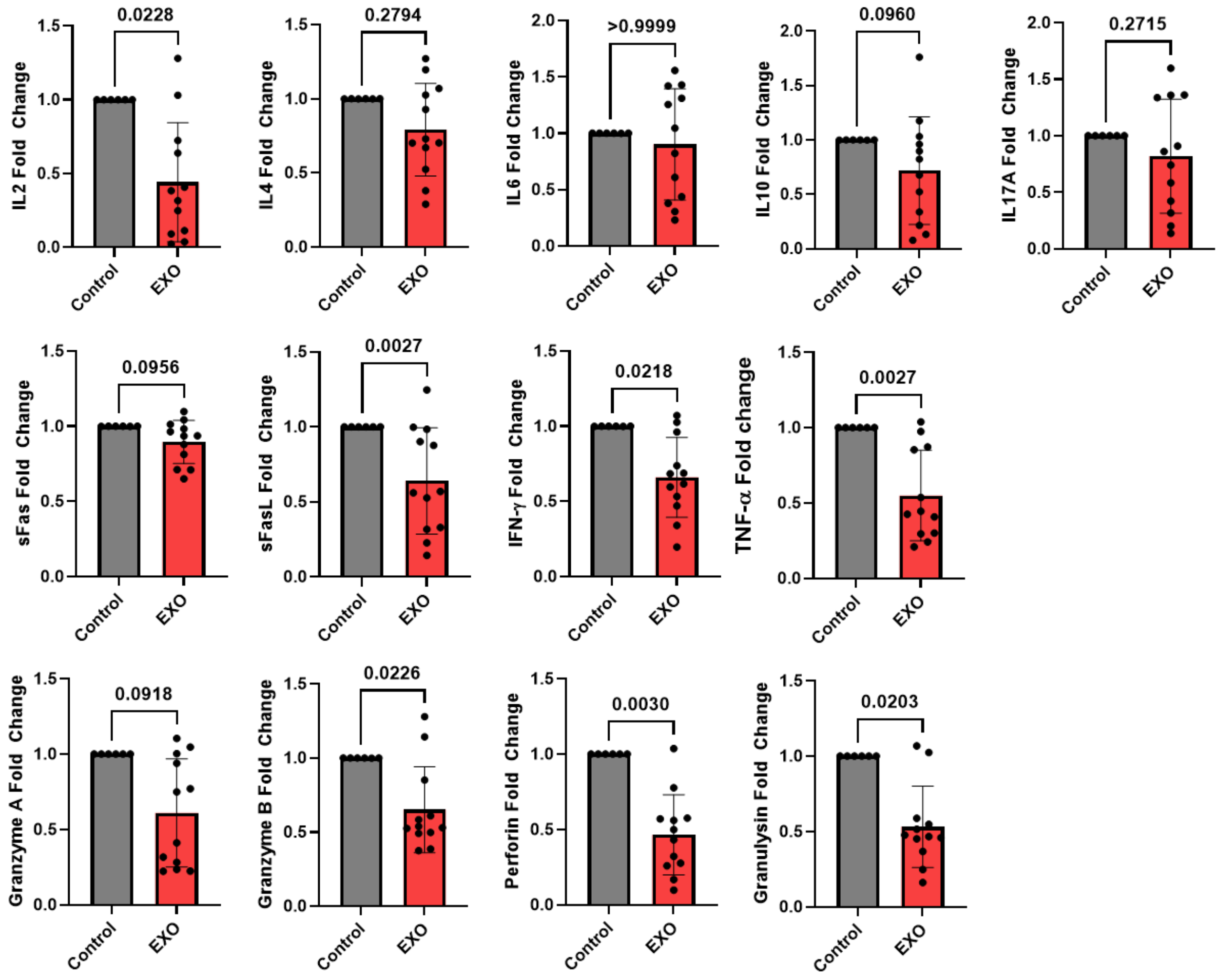

Fig. S3 Cytokine and cytotoxicity marker release profiles of activated CD8$^+$ T cells in the absence or presence of HNSCC exosomes. Multiplex cytokine release assay showing Fold change in the abundance of individual proteins in activated CD8$^+$ T cells that were treated with exosomes from HNC208, 285 and 365 (EXO, 0.5 X 10^9 particles/ ml) as compared to untreated controls. Data were normalized to protein levels in controls. Data were measured in n = 6 healthy donors (the same donors were used for both treatments). Statistical significance was determined by Mann-Whitney rank sum test.

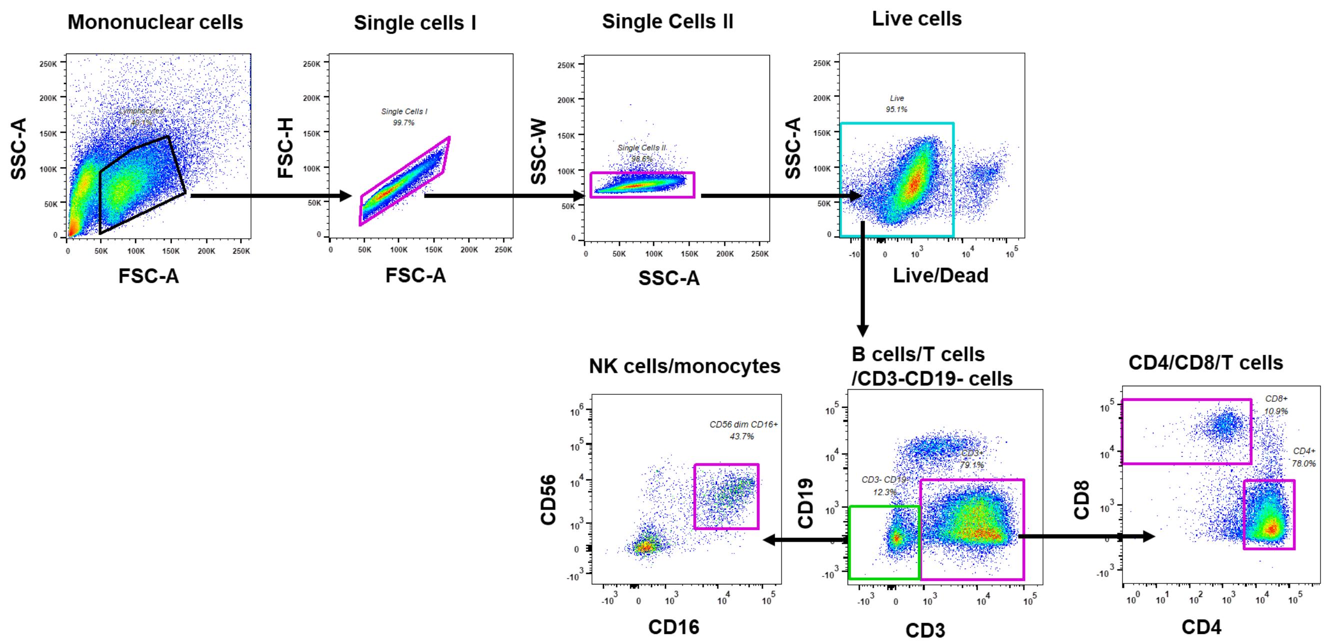

Fig. S4. Gating strategy for immune cell phenotyping flow cytometry experiments. Representative gating to identify various immune cell phenotypes from PBMCs from a single healthy donor is shown here. Briefly, out of the total events acquired, we first gated the mononuclear cell popular, followed by single cells and live cells (gated as Zombie UV^{+}). The live cell population was then gated as CD3-BV421^{+} T cells, CD19-APC-Cy7^{+} B cells or CD3^{-}CD19^{-} population. The CD3^{+} T cells were further classified into CD4-BV605^{+} and CD8-BV711^{+} subpopulations. The CD56-BV785 ^{dim} CD16^{-} NK cell population was identified within the CD3-CD19^{-} population. We then subdivided the monocyte subsets in the CD3^{-}CD19^{-} population based on the expression of CD16-APC and CD14-BV510. In all of the immune cell subsets that were defined as shown, we then measured the Kv1.3 (Alexa Fluor 555) and KCa3.1 (ATTO 488) abundance.

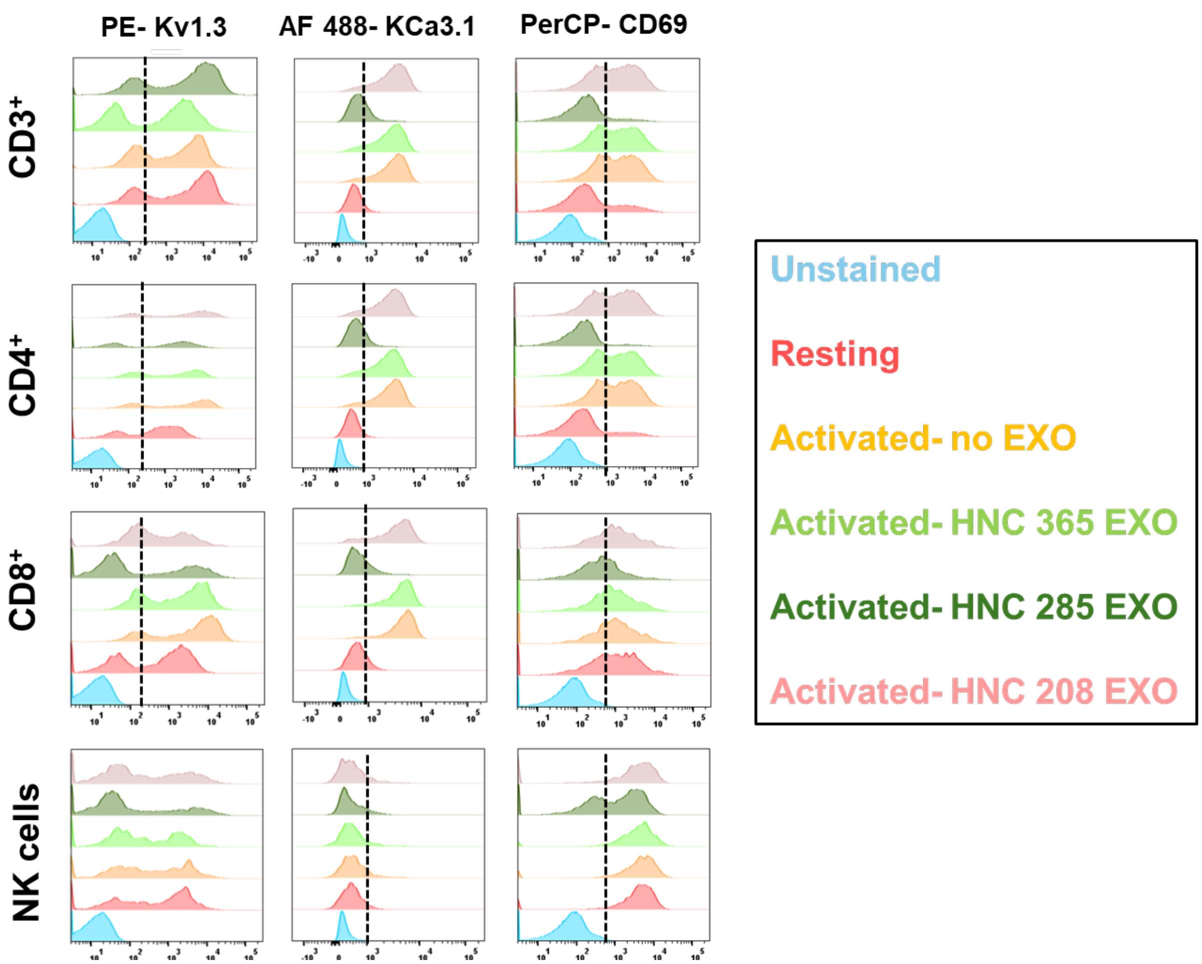

Fig. S5 Kv1.3, KCa3.1, CD69 expression in immune cell subsets in the absence or presence of HNSCC exosomes. Staggered histograms showing the abundance of Kv1.3, KCa3.1 and CD69 in an activated healthy donor PBMCs in the absence or presence of 1.04 X 10^9 particles/ ml HNSCC exosomes (EXO, derived from HNC208, HNC285 and HNC365 cells). Unstained cells and resting PBMCs were used as controls. Data from a representative healthy donor are shown.

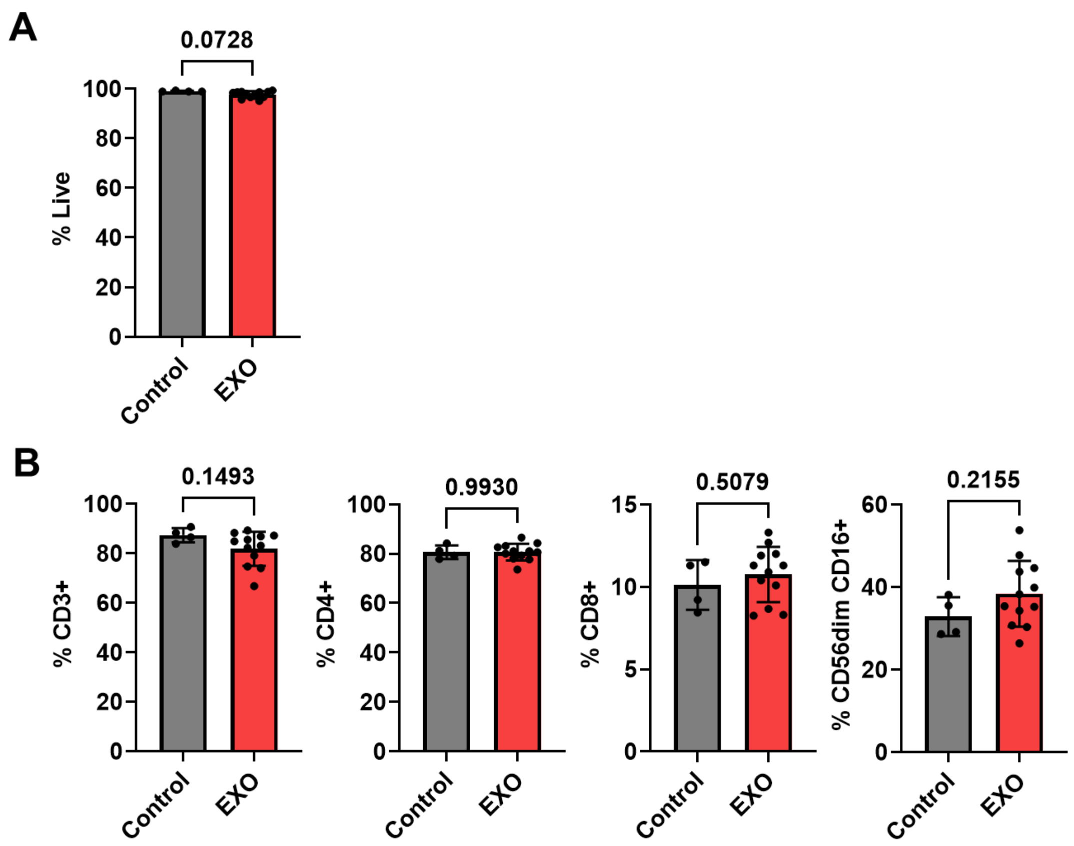

Fig. S6 Effect of HNSCC exosomes on PBMC viability and immune cell subset abundance
(A) Cell viability, measured by flow cytometry using Zombie UV live- dead stain and (B) Quantification of abundance of various immune cell subsets in four healthy donor PBMCs in the absence (control) or presence of HNC208, HNC285 and HNC365 exosomes (EXO, 1.04 X 10^9 particles/ ml) measured by flow cytometry. Some donors were treated with exosomes from multiple HNSCC patients. Untreated PBMC were used as controls. Significance determined by unpaired t-test. Bars represent means ± SD, symbols represent individual treatment conditions.

Table S1 Capture antibody panels to detect Immune Checkpoint proteins (Panel 1) and Tetraspanins (Panel 2) detected on surface of intact exosomes by multiplex ELISA

Panel 1				Panel 2		
**Spot #**	**Antibody**	**Vendor**	**Catalog #**	**Antibody**	**Vendor**	**Catalog #**
1	CD155	R&D systems	MAB25301	CD9	MSD	F215M
2	CD73	R&D systems	MAB5795	No antibody		
3	CD112	R&D systems	MAB2229	CD63	MSD	F215L
4	CD113	R&D systems	MAB3064	No antibody		
5	No antibody			No antibody		
6	No antibody			No antibody		
7	No antibody			No antibody		
8	CD39	R&D systems	MAB4397	No antibody		
9	PDL2	R&D systems	MAB12241	No antibody		
10	PDL1	MSD	B22Z7-2	CD81	MSD	F215N

Table S2: Marker genes for individual immune cells (NanoString cell type profiling).

Cell Type	Gene Name
B cells	BLK
	CD19
	MS4A1
	TNFRSF17
CD45	PTPRC
CD8 T cells	CD8A
	CD8B
Cytotoxic cells	CTSW
	GNLY
	GZMA
	GZMB
	GZMH
	KLRB1
	KLRD1
	KLRK1
	PRF1
DC	CCL13
	CD209
	HSD11B1
Exhausted CD8	CD244
	EOMES
	LAG3
Macrophages	CD163
	CD68
	CD84

Mast cells	MS4A2
	TPSAB1
Neutrophils	CSF3R
	FCGR3A
	S100A12
NK CD56^{dim} cells	IL21R
	KIR_Inhibiting_Subgroup_2
	KIR3DL1
	KIR3DL2
NK cells	NCR1
	XCL2
T cells	CD3D
	CD3E
	CD3G
	CD6
	SH2D1A
Th1 cells	TBX21
Treg	FOXP3

Table S3: Antibodies used for Flow Cytometry experiments

Marker	Fluorophore	Clone	Vendor	Catalog #	RRID
Kv1.3	N/A	N/A	Alomone	APC-101-GP	AB_2340958
IgG	Alexa-Fluor 555	N/A	ThermoFisher	A-21435	AB_2535856
KCa3.1	ATTO 488	6C1	Alomone	ALM-051	AB_10918529
CD19	APC-CY7	HIB19	Biolegend	302218	AB_314248
CD3	BV 421	UCHT1	Biolegend	300434	AB_10962690
CD4	BV 605	OKT4	Biolegend	317438	AB_11218995
CD8	BV 711	SK1	Biolegend	344734	AB_2565243
CD56	BV 785	HCD56	Biolegend	318348	AB_2563564
CD16	APC	3G8	Biolegend	302019	AB_492974
CD14	BV 510	63D3	Biolegend	367124	AB_2716229
CD69	PerCP Cy 5.5	FN50	Biolegend	310925	AB_2074957
Calmodulin	Alexa Fluor 647	2D1	Novus Biologicals	NB120-2860	-
Zombie UV	UV	N/A	Biolegend	423107	N/A

Table S4 List of differentially expressed genes

	Gene name	Gene family name	Immune Response Category	Annotation	p-value
1	AICDA	Apolipoprotein B mRNA editing enzyme catalytic subunits	T-Cell Functions	B-cell differentiation	6.33E-12
2	TPSAB1		Cell Functions	Basic cell functions, Cell Type specific	2.11E-10
3	CD14	Scavenger receptors| CD molecules		CD molecules, Innate immune response	3.05E-10
4	FUT7	Fucosyltransferases	Leukocyte Functions	Leukocyte migration	6.81E-10
5	IL1A	Endogenous ligands| Interleukins	Cytokines, Interleukins	Acute-phase response, Cytokines and receptors, Inflammatory response, Innate immune	2.48E-09

				response, Interleukins	
6	IL6	Interleukins| Interferons| Interleukin 6 cytokine family	Interleukins	Humoral immune response, Interleukins	1.27E-08
7	TNFSF14	Tumor necrosis factor superfamily| CD molecules	Cytokines, Regulation, T-Cell Functions, TNF Superfamily	CD molecules, Cytokines and receptors, Regulators of T-cell activation, TNF superfamily members and their receptors, T-cell regulators, T-cell proliferation	3.81E-08
8	IL3	Interleukins	Regulation, T-Cell Functions	Interleukins, Regulators of Th1 and Th2 development	1.02E-07

9	APOE	Apolipoproteins	Transporter Functions	Cell Type specific, Lipid transporter activity	1.24E-07
10	CXCL3	Endogenous ligands| Chemokine ligands	Chemokines, Regulation	Chemokines and receptors, Regulation of inflammatory response	1.56E-07
11	IL23A	Interleukins	Interleukins	Innate immune response, Interleukins	2.24E-07
12	HLA-DQA1	C1-set domain containing| Histocompatibility complex	Antigen Processing	Adaptive immune response, Antigen processing and presentation	4.04E-07
13	SLC11A1	Solute carriers	Macrophage Functions	T-cell proliferation	4.65E-07
14	CXCL2	Endogenous ligands| Chemokine ligands	Chemokines, Regulation	Regulation of inflammatory response, Chemokines and receptors	4.92E-07

15	CREB5	Basic leucine zipper proteins		Complement pathway, Inflammatory response	6.67E-07
16	CX3CR1	C-X-3-C motif chemokine receptors	Chemokines, Microglial Functions	Adaptive immune response, Chemokines and receptors, Microglial cell activation	7.16E-07
17	SH2D1B	SH2 domain containing	Leukocyte Functions	Leukocyte activation	1.55E-06
18	SERPINB2	Serpin peptidase inhibitors	Senescence	Senescence initiators interferon related	1.79E-06
19	THBD	CD molecules| C-type lectin domain containing	Leukocyte Functions	CD molecules, Leukocyte migration	2.17E-06
20	LILRB2	Inhibitory leukocyte immunoglobulin like receptors| CD molecules| Ig-like cell adhesion molecule family	Regulation	CD molecules, Regulation of immune response	4.33E-06

21	CCL18	Chemokine ligands	Chemokines	Chemokines and receptors, Anti-inflammatory cytokines	9.50E-06
22	CD276	C2-set domain containing| CD molecules| V-set domain containing	Regulation	CD molecules, Regulation of immune response	1.13E-05
23	IL9	Interleukins	Cytokines	Cytokines and receptors, Interleukins, Regulation of inflammatory response	2.43E-05
24	TLR4	CD molecules| Toll like receptors	Microglial Functions, TLR	CD molecules, Innate immune response, Microglial cell activation, Toll-like receptor	3.40E-05

25	KLRG1	Killer cell lectin like receptors| C-type lectin domain containing	NK Cell Functions, Regulation	Innate immune response, NK cell functions, Regulation of immune response	3.99E-05
26	LILRB3	Inhibitory leukocyte immunoglobulin like receptors| CD molecules	Regulation	CD molecules, Regulation of immune response	5.12E-05
27	PLAU		Senescence	Senescence pathway	5.51E-05
28	NLRP3	NLR family| Pyrin domain containing		Innate immune response	6.43E-05
29	IL23R		Cytokines	Cytokines and receptors	6.85E-05
30	CCL8	Endogenous ligands| Chemokine ligands	Chemokines, Regulation	Chemokines and receptors, Regulation of inflammatory response	6.98E-05

31	MSR1	Scavenger receptors| Scavenger receptor cysteine rich domain containing| CD molecules	Cell Functions	Basic cell functions, Cell Type specific, CD molecules	7.54E-05
32	CCL20	Endogenous ligands| Chemokine ligands	Chemokines	Chemokines and receptors	8.46E-05
33	MRC1	Scavenger receptors| CD molecules| C-type lectin domain containing		CD molecules	0.00016607
34	THBS1		Antigen Processing, Cell Cycle, Regulation	Cell cycle arrest, Cell Type specific, Chronic inflammatory response, Negative regulation of antigen processing	0.00017981
35	LILRA1	Activating leukocyte immunoglobulin like receptors| CD molecules	Regulation	CD molecules, Regulation of immune response	0.00018715
36	AXL	Immunoglobulin like domain containing| V-set domain containing| Fibronectin type III		Innate immune response	0.00023864

		domain containing| Receptor Tyrosine Kinases			
37	NRP1	CD molecules	Cell Functions	Basic cell functions, CD molecules	0.00025617
38	CD36	Scavenger receptors| CD molecules	Transporter Functions	CD molecules, Receptors involved in phagocytosis	0.00039933
39	CX3CL1	Endogenous ligands| Chemokine ligands	Chemokines, Leukocyte Functions	Adaptive immune response, Leukocyte activation, Chemokines and receptors, Th1 orientation	0.00042197
40	CMKLR1	Chemerin receptor	Chemokines	Chemokines and receptors	0.00043841
41	IL11	Endogenous ligands| Interleukins| Interleukin 6 cytokine family	B-Cell Functions, Cytokines, Interleukins,	Anti-inflammatory cytokines, B-cell	0.00049627

			T-Cell Functions	differentiation, Interleukins	
42	TLR8	CD molecules| Toll like receptors	TLR	CD molecules, Innate immune response, Toll-like receptor	0.00051752
43	FOXJ1	Forkhead boxes		Cell Type specific, Humoral immune response	0.00055677
44	CD160	Immunoglobulin like domain containing| CD molecules	Regulation	Cell Type specific, CD molecules, Regulation of immune response	0.00066123
45	ITGB3	Integrin beta subunits| CD molecules	Adhesion	Adhesion, CD molecules	0.00075606
46	CLEC7A	Scavenger receptors| CD molecules| C-type lectin domain containing		Innate immune response	0.00092245
47	HLA-DQB1	C1-set domain containing| Histocompatibility complex	Antigen Processing	Adaptive immune response,	0.00121075

				Antigen processing and presentation	
48	BST1	CD molecules		CD molecules, Humoral immune response	0.00129477
49	C1QA	Complement system	Complement	Complement pathway, Innate immune response	0.00150679
50	CFI	Scavenger receptor cysteine rich domain containing| Complement system		Innate immune response	0.00156695
51	MARCO	Scavenger receptors| Scavenger receptor cysteine rich domain containing		Cell Type specific, Innate immune response	0.00169533
52	KIR_Activating_Subgroup_1		NK Cell Functions, Regulation	CD molecules, NK cell functions, Regulation of	0.00195088

				immune response	
53	EOMES	T-boxes	T-Cell Functions	CD8-positive, T-cell differentiation	0.00239442
54	AMBP	Lipocalins	Regulation	Negative regulation of immune response	0.0031724
55	CD163	Scavenger receptors| Scavenger receptor cysteine rich domain containing| CD molecules	Transporter Functions	CD molecules, Phagocytosis	0.00329129
56	CLEC5A	C-type lectin domain containing		Innate immune response	0.00550031
57	CSF1R	Immunoglobulin like domain containing| CD molecules| Receptor Tyrosine Kinases		CD molecules, Innate immune response	0.01109937
58	TREM1	CD molecules| V-set domain containing		CD molecules, Humoral immune response	0.01151486

59	CXCR2	CD molecules| C-X-C motif chemokine receptors| Interleukin receptors	Chemokines, Regulation	CD molecules, Chemokines and receptors, Regulation of inflammatory response	0.01255581
60	CD209	Scavenger receptors| CD molecules| C-type lectin domain containing	DC	Basic cell functions, Cell Type specific, CD molecules	0.0164046
61	MPPED1		Cell Functions	Basic cell functions, Cell Type specific	0.02375641
62	IL34	Interleukins	Interleukins	Innate immune response, Interleukins	0.02628454
63	CCR9	CD molecules| C-C motif chemokine receptors		CD molecules, Innate immune response	0.03552989
